# Risk factors affecting the utilization of eye care services evaluated by the CDC's behavior risk factor surveillance system from 2018 to 2021

**DOI:** 10.3389/fpubh.2024.1335427

**Published:** 2024-06-10

**Authors:** Adrianna M. Powers, Deepkumar Patel, Margaret M. DeAngelis, Changyong Feng, Karen Allison

**Affiliations:** ^1^New York Medical College, Valhalla, NY, United States; ^2^University of San Francisco, San Francisco, CA, United States; ^3^University at Buffalo, Buffalo, NY, United States; ^4^University of Rochester, Rochester, NY, United States

**Keywords:** diabetic retinopathy, glaucoma, eye care utilization, quality of life, health disparities, risk factors

## Abstract

When thinking about major health concerns in the U.S. and around the world, eye care ranks lower compared to cardiovascular disease, cancer, and diabetes. However, people do not think about the direct connection between diabetes and eye health. Untreated diabetes can lead to visual impairments such as blindness or difficulty seeing. Studies have found that eye health associated with nutrition, occupational exposure, diabetes, high blood pressure, and heart disease are some of the known risk factors. This study aimed to identify the potential risk factors that are associated with visual impairment (VI). The data used for this analysis were obtained from the Centers for Disease Control and Prevention (CDC) - Behavioral Risk Factor Surveillance System (BRFSS) from 2018 to 2021. We found important characteristics, such as the U.S. region, general health perception, employment status, income status, age, and health insurance source, that are associated with VI. Our study confirmed that the common demographical factors including age, race/ethnicity, the U.S. region, and gender are associated with VI. The study also highlights associations with additional risk factors such as health insurance source, general health perceptions, employment status, and income status. Using this information, we can reach out to communities with large numbers of individuals experiencing vision challenges and help educate them on prevention and treatment protocols, thereby effectively addressing VI and blindness challenges within our communities, neighborhoods, and finally, the broader society.

## Introduction

Visual impairment (VI) is a worldwide issue that greatly impacts the quality of life and decreases life expectancy in the aging population (1). According to Assi et al. (2), a billion people worldwide struggle with VI and blindness although these conditions can be prevented or slowed with treatment. If we educate the public on the conditions that can make a person more susceptible to vision loss and the treatments that can prevent or slow their progression, this may result in blindness and decreasing rates of VI (3). The risk factors involved in influencing VI or blindness can be socioeconomic, biological, or demographic (4).

This study focuses on ophthalmological health and the utilization of eye health care. Eye care has been neglected as a routine health procedure over the years. People neither understand nor are educated on the importance of an annual eye examination, especially when they have diseases, such as diabetes, which can greatly affect vision. According to Besagar et al. (5), “visual impairment is one of the most common disabilities in the U.S., with ~32 million U.S. adults exhibiting blindness or other difficulty seeing despite use of glasses or contact lenses.” VI is becoming more prevalent, yet this trend has largely been ignored. The risk factors that can lead to VI include socioeconomic status (SES), genetics, accessibility to health care, and other diseases/comorbidities. When someone becomes visually impaired, their everyday life is greatly affected. They may lose their independence and a sense of purpose, leading to a decrease in their overall mental and physical health (5). Previous studies have shown that those who are in low SES have a substantially higher disease burden (6). Individuals with low SES are characterized by having low levels of education, income, and occupational status. These attributes are commonly found among marginalized groups. Being part of the low SES class also entails limited access to healthcare and a lack of knowledge regarding health-related issues. Since these individuals rely on state insurance, their options for doctors and medical care are restricted. Additionally, due to financial constraints, they often cannot afford to consult a doctor or purchase medication when they fall ill. In contrast, individuals in high SES groups have a greater awareness of the importance of eye health (3). These findings demonstrate that health disparities play a huge role in the level of health literacy and overall understanding of health care. In a recent study conducted by the American Academy of Ophthalmology (AAO), a mere 19% of respondents could name the three main causes of blindness, which are diabetic retinopathy, glaucoma, and age-related macular degeneration (3). Social determinants of health (SDOH) encompass various factors, such as economic stability, educational access and quality, access to quality healthcare, a safe neighborhood and built environment, and the overall social community, which have also shown a relationship with overall health. These determinants disproportionately impact individuals from lower SES and marginalized groups, who often face multiple challenges in these areas. The impact of SDOH can vary across states, with some states being more affected than others based on the level of government support in implementing beneficial programs. Individuals with a low SES face challenges in accessing adequate healthcare due to a lack of insurance or being underinsured. Consequently, they are more vulnerable to experiencing advanced stages and complications of diseases such as diabetes and glaucoma. A synopsis of the three main causes of blindness will be described in the following paragraph.

Diabetic retinopathy (DR) affects a third of those who have been diagnosed with diabetes, and the prevalence of DR continues to grow as the duration of diabetes lengthens (7). DR affects about 8 million Americans yet only 50% of people with diabetes receive the necessary annual eye examination (8). Two major risk factors for the development of DR are hyperglycemia and hypertension (7). DR is asymptomatic in its early stage and can be detected via eye dilation and retinal evaluation during an annual eye examination (9), which helps diagnose DR at the early stage and decreases the number of people who become blind by 94% (8). In the working-age population, 75% of people with type 1 diabetes and 50% of people with type 2 diabetes will develop DR (9). In the aging population, retinal changes, such as a decrease in blood flow, retinal thinning, and microglial changes, can make them more vulnerable to severe retinal damage from oxidative and ischemia changes (9). The prevalence of DR in adults with diabetes in the U.S. is 29% (7). In the U.S., the 10-year incidence of retinopathy is 74% at the baseline, 64% develop severe retinopathy, and 17% develop proliferative DR (7). Fairless et al. indicated that ethnicity and sociodemographic elements play a significant role in developing diabetic retinopathy.

Glaucoma is a chronic progressive optic neuropathy that causes damage to the optic nerve head (ONH) and the retinal nerve fiber layer (RNFL) (10). The damage is caused by increased pressure in the eye leading to VI and blindness. Approximately 3 million people in the U.S. have glaucoma, making it a major cause of irreversible vision loss in the U.S. (10). Many people have a very general idea of what glaucoma is but are not aware of its causes. Genetics, immune system disorders (sarcoidosis, rheumatoid arthritis, and lupus), certain infectious diseases (herpes and toxoplasmosis), endocrine disorders (diabetes), steroids, and low blood pressure are all factors contributing to the development or progression of glaucoma (10). Glaucoma can be treated, and its progression can be restricted when detected early. Annual eye examinations can facilitate the early detection of glaucoma. During eye exams, the IOP is measured, gonioscopy is performed, and perimetry and other testing are conducted to further monitor the disease (10). A previous study found that in 2017, 3,973,400 people were diagnosed with glaucoma; of this, 16,200 people were between the ages of 18–39 years; 235,100 were between the ages of 40–64 years; 3,000,300 were between the ages of 65–84 years; and 721,900 were 85 years and older (11). The crude annual prevalences of glaucoma diagnosis by race/ethnicity were as follows: 29.76% black non-Hispanics, 22.74% Asians, 19.80% Hispanics, 18.04% North American Natives, and 17.50% white non-Hispanics (11). The literature also noted the glaucoma prevalence in blacks was 6.1% compared to 2.8% in whites (12).

Age-related macular degeneration (ARMD) is a major cause of irreversible VI in older adult people; in the early stages, it is asymptomatic, while in later stages, severe vision loss frequently occurs (13). The prevalence of early ARMD to progress late ARMD is 5% over a 5-year period with an increase of 15% over a 15-year period (13). ARMD has multiple risk factors such as age, ocular dysfunction, systemic diseases, diet, smoking, genetics, and environmental factors (13). Advanced ARMD is considered rare before the age of 55 years and is more common in those who are 75 years and older (14).

Both glaucoma and diabetic retinopathy are eye diseases that can be prevented and/or successfully treated when detected early, thereby reducing the amount of vision loss in patients. There are many barriers to people seeking necessary health care and most do not seek care until their health starts to decline and changes become alarming For most diseases/illnesses, precautionary screening is not attainable or is not sought due to factors such as cost, insurance status, poor patient-physician communication, lack of trust, absence of symptoms, and no perceived need for examination (8). These factors are among the various barriers that interfere with people seeking care. Fairless and Nwanyanwu (8) categorized these barriers into seven categories: vision status (noticeable changes in vision before the person seeks an eye exam), competing concerns (employment schedule, childcare, and other health issues), emotional context (fear of negative news or procedures), resource availability (insurance coverage, cost, and transportation), in-clinic experiences (patient-physician interactions), cues to action (primary care referrals to other health providers and appointment reminders), and knowledge-creating experiences (information about diseases/illnesses from doctor, family, and friends, as well as misinformation). In 2020–2021, another barrier was the COVID-19 pandemic, which impacted the availability and delivery of healthcare (15). Ophthalmologic diagnosis relies heavily on physical examinations and imaging that cannot be performed via telemedicine which has become the new way of seeking health care during the pandemic (15). These barriers significantly contribute to the steady increase in several illnesses and diseases being experienced today.

The purpose of this study was to examine the potential risk factors of those who are VI using the BRFSS web analysis tool from 2018 to 2021. The BRFSS is a health-related telephone survey that collects data about U.S. residents regarding their health-related risk behaviors, chronic health conditions, and the use of prevention services. It collects data from all 50 states including the District of Columbia and the three U.S. territories (16). We will examine demographics, socioeconomic status, a personal reflection on health, insurance status, and several other characteristic traits. Other studies have suggested that these characteristics need to be further examined to observe which factors are more associated with visual impairment. Learning about these characteristics helps in the tailoring of interventions and education-based learning for patients and physicians.

## Methods

The data used for this study were obtained from CDC's BRFSS web-enabled analysis tool from 2018 to 2021. This database is a health-related telephone survey that collects data about U.S. residents (approximately 400,000 random interviews in a year). A list of all U.S. telephone numbers, both landlines and cellular lines, is placed into a system that randomly picks a phone number to call (17). The phone interviews lasted for about 25 min per call. It collects data from U.S. residents related to health-related risk behaviors, chronic health conditions, and the use of preventive services (17). The full protocol for the BRFSS can be found on the CDC's website which will explain each step they took to develop the data used in this study (17). From the phone interview questionnaire, we specified variables of interest in the categories of demographic information, socioeconomic status, eye care health, and diabetic information after going through all the questions asked in the questionnaire. This includes variables such as “sex,” “race/ethnicity,” “[in the] past year needed to see a doctor but could not because of cost,” “how long has it been since you visited a doctor for a routine checkup,” “level of education,” “employment status,” “annual income,” “type of insurance coverage,” “ten-level age category,” “three-categories of body mass index,” “language identifier,” “region,” and “region classification.” These variables were chosen based on the literature review and previous knowledge of the topic. From this, we identified the variables that previous studies have found significant and then chose other variables that may also be associated with eye care utilization.

**Figure 1:**
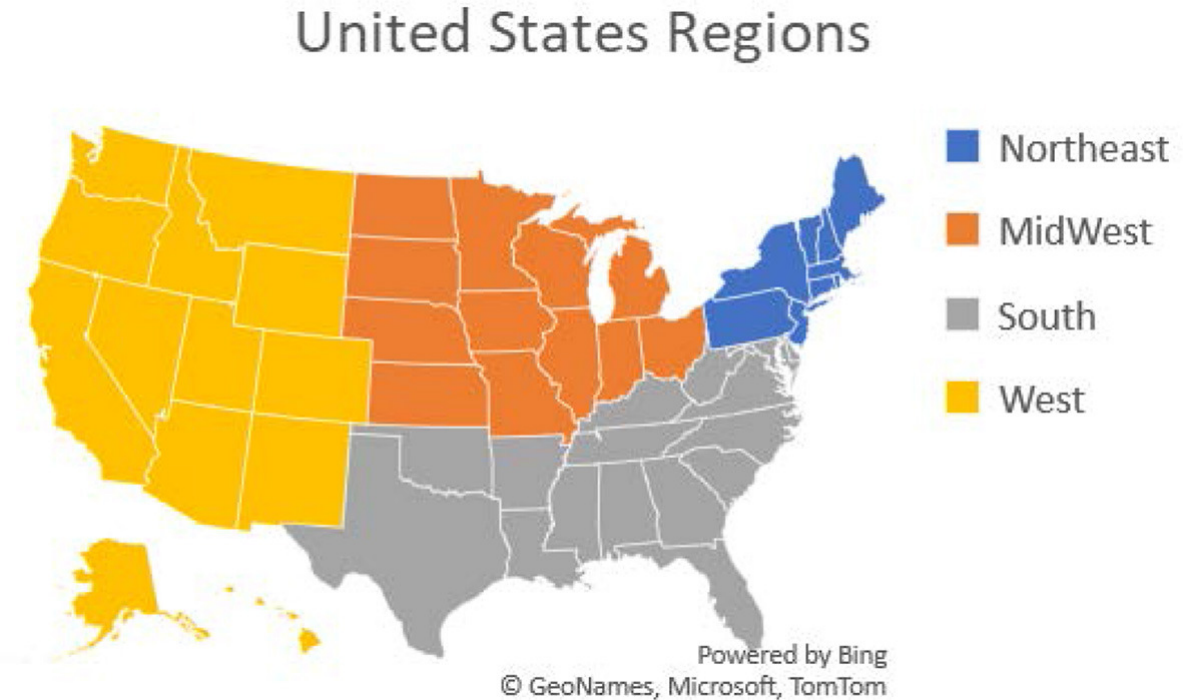
United States regions: the Northeast, Midwest, South, and West. Puerto Rico and Guam were also included.

We then had to do some variable modifications and group the variables to allow for clear analysis. All modifications were made with scientific proof of validity. For example, the states were listed by name. We classified them into four regions, namely, Northeast, Midwest, South, and West as shown in Figure 1. This classification enabled us to determine whether the geographical location is associated with VI and can later aid in targeting these areas for intervention and prevention strategies. Once all variables were ready for analysis, descriptive analysis was performed.

Descriptive analysis included 27 variables as follows: “region,” “sex,” “private residency,” “general health,” “physical health,” “health care coverage,” “personal doctor,” “medical cost,” “checkups,” “blood pressure medication,” “diabetes,” “education level,” “housing status,” “employment,” “income level,” “blindness,” “high blood pressure/ diabetes testing,” “diabetes/retinopathy,” “pre-diabetic,” “visit diabetes doctor,” “type of insurance coverage,” “race/ethnicity,” “provider of majority of care,” “age,” “BMI,” “language,” and “region classification” for each year from 2018 to 2021. These 27 variables were selected as variables of interest for further analysis to find an association with VI.

Next, we performed a weighted bivariate analysis to examine the association between the response variable (Blindness “are you blind or have serious difficulties seeing, even when wearing glasses?”) and the selected 17 explanatory variables from the 27 variables of the descriptive analysis: “region,” “sex,” “general health,” “personal doctor,” “medical cost,” “checkups,” “blood pressure medication,” “diabetes,” “education level,” “employment,” “income level,” “type of insurance coverage,” “race/ethnicity,” “age,” “BMI,” “language,” and “region classification.” These variables were selected based on significance levels and confidence intervals. There was a weighted total of 6,269,897 individuals who responded “yes” that they are blind or have severe difficulties seeing over the 4 years. The final step of this study was a multiple logistic regression analysis using PROC SURVEYLOGISTIC. In this step, reference groups were chosen for each explanatory variable. This was accomplished by taking the response/category with the lowest total percentage from each explanatory variable and assigning it as the reference group. This multiple logistic regression analysis was used to study the association between the occurrence of the disease (responding “yes” to the blindness variable) and the demographic and clinical risk factors (explanatory variables). The significance level was set at 0.05. All analyses were implemented with SAS 9.4 (SAS Institute, Inc., Cary, NC).

## Results

Variables responses were classified into specific categories per variable for analysis purposes. Recoding was performed to reduce the varied responses into general response categories (Table 1). This enables a more accurate analysis of each explanatory variable. The responses that were missing (“.”) or classified as “do not know/ not sure,” “refused,” or “blank” were not used in the analysis. All data were weighted to account for these missing responses.

**Table 1:** The variables are chosen for the analysis with their assigned codes for analysis purposes.

**Variable**	**Question**	**Categories in dataset**	**Categories desired for analysis**
State/region	State federal information processing standard (fip) code	States listed with #: 1, 2, 4, 5, 6, 8, 9, 10, 11, 12, 13, 15, 16, 17, 18, 19, 20, 21, 22, 23, 24, 25, 26, 27, 28, 29, 30, 31, 32, 33, 34, 35, 36, 37, 38, 39, 40, 41, 42, 44, 45, 46, 47, 48, 49, 50, 51, 53, 54, 55, 56, 66, 72, 78	1 = Northeast (9, 23, 25, 33, 34, 36, 42, 44, 50) 2 = Midwest (17, 18, 19, 20, 26, 27, 29, 31, 38, 39, 46, 55) 3= south (1, 5, 10, 11, 12, 13, 21, 22, 24, 28, 37, 40, 45, 47, 48, 51, 54) 4 = west (2, 4, 6, 8, 15, 16, 30, 32, 35, 41, 49, 53, 56) 0 = guam, puerto rico, and virgin islands (66,72,78)
Sex	What is your sex?	1 = male 2 = female 3 = other 7 = do not know/ not sure 9 = refused	1 = male 0 = female
General health	Would you say that in general, your health is	1 - excellent 2 - very good 3 - good 4 - fair 5 - poor 7 – do not know/not sure 9 - refused Blank - not asked/missing	1 = excellent, very good 2 = good health 0 = fair, poor
Health care coverage	Do you have any kind of health care coverage, including health insurance, prepaid plans, such as Health Maintenance Organization (hmos), or government plans, such as Medicare and Medicaid, or Indian Health Services?	1 - yes 2 - no 7 – do not know/not sure 9 - refused Blank - not asked/missing	1 = yes 0 = no
Personal doctor	Do you have one person you think of as your personal doctor or health care provider?	1 - yes, only one 2 - more than one 3 - no 7 – do not know/not sure 9 - refused Blank - not asked/missing	1 = yes, one or more doctors 0 = no
Medical cost	Was there a time in the past 12 months when you needed to see a doctor but could not because of cost?	1 - yes 2 - no 7 – do not know/not sure 9 - refused Blank - not asked/missing	1 = yes 0 = no
Checkups	About how long has it been since you visited a doctor for a routine checkup?	1 - within the past year 2 - within the past 2 years 3 - within the past 5 years 4 - 5+ years ago 7 – do not know/not sure 8 - never 9 - refused Blank - not asked/missing	1 = past month/year 0 = past 5 or more years
Hypertension	Are you currently taking medication for high blood pressure?	1 - yes 2 - no 7 – do not know/not sure 9 - refused Blank - not asked/missing	1 = yes 0 = no
Diabetes	(Ever told) you have diabetes	1 - yes 2 - yes, but the female said that during pregnancy 3 - no 4 - no, prediabetes or borderline diabetes 7 – do not know/not sure 9 - refused Blank - not asked/missing	1 = yes 0 = no
Education level	What is the highest grade or year of school you completed?	1 - never attended or only kindergarten 2 - grades 1 through 8 3 - grades 9 through 11 4 - grade 12 or GED 5 - college 1 year through 3 years 6 - college 4 years or more 9 - refused Blank - not asked/missing	1 = less than high school 2 = graduated high school 3 = attended college 4 = graduated college
Employment	Are you currently..?	1 - employed for wages 2 - self-employed 3 - out of work for 1 year or more 4 - out of work for less than 1 year 5 - a homemaker 6 - a student 7 - retired 8 - unable to work 9 - refused Blank - not asked/missing	1 = employed 2 = unemployed 3 = homemaker 4 = student 5 = retired 6 = unable to work
Income levels	Is your annual household income from all sources:	1 - < USD 10,000 2 - < USD 15,000 3 - < USD 20,000 4 - < USD 25,000 5 - < USD 35,000 6 - < USD 50,000 7 - < USD 75,000 8 - USD 75,000 or more 77 – do not know/ not sure 99 - refused Blank - not asked/missing	1 = < USD 35,000 2 = USD 35,000–74,999 3 = more than USD 75,000
Blindness	Are you blind or do you have serious difficulty seeing, even when wearing glasses?	1 - yes 2 - no 7 – do not know/not sure 9 - refused Blank - not asked/missing	1 = yes 0 = no
Type of insurance coverage	What is the primary source of your health care coverage? Is it…	1 - plan through employer/union 2 - plan that you or another family member buys 3 - medicare 4 - medicaid or other state programs 5 - TRICARE, Veterans Affairs, and military 6 - Alaskan Native, Indian health service, and tribal health services 7 - some other source 8 - none 77 – do not know/not sure 99 - refused Blank - not asked/missing	1 = through employer 2 = personal plan 3 = state program 4 = military 5 = other
Race/ ethnicity	Imputed race/ethnicity value	1 - white, non-Hispanic 2 - black, non-Hispanic 3 - Asian, non-Hispanic 4 - American Indian/Alaskan Native, non-Hispanic 5 - Hispanic 6 - other races, non-Hispanic	1 = white, non-Hispanic 2 = black, non-Hispanic 3 = Hispanic 0 = other
Age	14-level age categories	1 - aged from 18 to 24 2 - aged from 25 to 29 3 - aged from 30 to 34 4 - aged from 35 to 39 5 - aged from 40 to 44 6 - aged from 45 to 49 7 - aged from 50 to 54 8 - aged from 55 to 59 9 - aged from 60 to 64 10 - aged from 65 to 69 11 - aged from 70 to 74 12 - aged from 75 to 79 13 - aged 80 or older 14 – do not know/ refused/missing	1 = 40–44 years old 2 = 45–49 years old 3 = 50–54 years old 4 = 55–59 years old 5 = 60–64 years old 6 = 65–69 years old 7 = 70–74 years old 8= 75–79 years old 9 = 80+ years old 0 = < 40 years old
BMI	Four categories of body mass index	1 – underweight (BMI < 5th percentile) 2 - normal weight (5th percentile < = BMI < 85th percentile) 3 - overweight (85th percentile < = BMI < 95th percentile) 4 – obese (BMI >= 95th percentile) Blank- do not know/ refused/ missing	1 = underweight 2 = normal weight 3 = overweight/obese
Language	Language identifier	1 = English 2 = Spanish 3–99 = other	1 = English 0 = Spanish
Region classification	Region	1 = urban 2= rural	1 = urban 0 = rural

The BMI categories are based on the CDC's categorization that is age and gender-specific.

The main insurance plans are defined as HMOs, PPOs, Medicare, and Medicaid. HMOs provide care through an approved network of doctors and require referrals for specialist doctors. They provide cheaper care with co-pays. However, the only downside is that out-of-network care will not be covered and everything will have to be paid out of pocket. In a PPO, you can see doctors who are in-network or out-of-network, and referrals are not needed. The downside is the cost of coverage which is more expensive than HMO plans. Medicare plans are federal health insurance for anyone over 65 years of age and those who are under 65 years with disabilities and certain conditions. Medicaid is a joint federal and state program that provides some health coverage to those who have low income and limited resources. Income qualifications depend on the number of people in the household and the state you reside in.

An analysis of the yearly data revealed that the highest percentage of those with VI had the following characteristics: they were from the South, female, lived in a private residence, reported their health as excellent or very good, had health insurance, had a primary care physician and had routinely visited them, were white non-Hispanic, were < 40 years, had hypertension, etc. The results of the descriptive analysis are shown in Table 2. This gives an overview of the general characteristics of the surveyed population for each year.

**Table 2:** Univariate analysis of 27 selected variables from the yearly BRFSS datasets.

**Descriptive analysis of each variable**									
		**Year 2018**		**Year 2019**		**Year 2020**		**Year 2021**	
**Variable**		**Weighted frequency**	**Percent**	**Weighted frequency**	**Percent**	**Weighted frequency**	**Percent**	**Weighted frequency**	**Percent**
Region	Guam and Puerto Rico	932,151	2.66	874,602	3.08	982,698	2.76	904,930	3.72
	Northeast	5,020,880	14.31	3,662,703	12.92	5,390,689	15.12	3,628,617	14.91
	Midwest	8,180,076	23.31	6,548,514	23.09	7,160,230	20.08	5,133,029	21.09
	South	12,681,643	36.14	10,204,953	35.99	11,643,231	32.65	7,327,319	30.1
	West	8,271,581	23.57	7,066,440	24.92	10,487,563	29.41	7,347,019	30.18
Sex	Male	16,688,903	47.67	13,485,224	47.55	17,043,091	47.79	11,707,118	48.1
	Female	18,322,938	52.33	14,871,987	52.45	18,621,321	52.21	12,633,795	51.9
Private residency	Yes	26,934,038	99.45	22,231,612	99.56	28,648,872	99.58	19,964,549	99.58
	No	148,186	0.55	97,582	0.44	120,015	0.417	84,237	0.42
General health	Excellent/ very good	17440893	49.83	13887471	49.09	20115955	56.52	12,684,965	52.23
	Good	11,057,040	31.59	9,000,998	31.81	10,329,572	29.02	7,614,492	31.36
	Fair/poor	6,505,269	18.58	5,403,937	19.1	5,146,077	14.46	3,985,216	16.41
Physical health	One or more days of physically unhealthy	12,643,464	36.04	10,387,049	36.63	9,866,516	27.66	7,705,310	31.66
	No. days of physically unhealthy	22,442,867	63.96	17,970,162	63.37	25,797,896	72.34	16,635,603	68.34
Health care coverage	Yes	31,412,808	89.94	25,214,888	89.34	31,982,797	90.11	21,858,242	93.12
	No	3,512,524	10.57	3,007,296	10.66	3,509,580	9.89	1615760	6.88
Personal doctor	Yes, one or more providers	27,846,983	79.86	22,525,029	79.81	28,192,581	79.46	20,605,467	85.39
	No providers	7,024,489	20.14	5,697,952	20.19	7,285,537	20.54	3,526,172	14.61
Medical cost	Yes, the cost stopped me from seeing a doctor	4,183,786	11.98	3,494,096	12.35	3,428,567	9.64	2,143,989	8.83
	No cost has not stopped me	30,747,952	88.02	24,787,186	87.65	32,149,947	90.36	22,126,009	91.17
Checkups	Within the past 2 year	30,784,216	89.4	24,963,834	89.57	31,410,602	89.59	21,293,014	89.14
	Within the past 5+ years or never	3,649,613	10.6	2,905,854	10.43	3,649,868	10.41	2,593,966	10.86
Blood pressure meds	Yes			8,158,078	79.58			6,978,901	80.52
	No			2,093,590	20.42			1,688,434	19.48
Diabetes	Yes, I have diabetes	4,705,499	13.44	3,783,200	13.37	4,478,324	12.58	3,216,587	13.24
	No, I do not have diabetes	30,316,423	86.56	24,517,615	86.63	31,113,520	87.42	21,072,074	86.76
Education level	Less than high school	1,119,418	3.2	1,011,138	3.58	1,053,100	2.97	84,4407	3.49
	Graduated high school	10,457,595	29.92	8,276,293	29.32	9,957,358	28.06	6,628,012	27.38
	Attended college	9,455,757	27.05	7,599,214	26.92	9,382,514	26.44	6,213,126	25.67
	Graduated college	13,918,884	39.82	11,342,942	40.18	15,090,952	42.53	10,518,491	43.46
Housing status	Rent	22,273,809	63.95	18,061,579	64.23	22,157,251	62.71	15,305,106	63.56
	Own	10,524,532	30.22	8,441,570	30.02	11,085,940	31.38	7,399,526	30.73
	Other arrangements	2,029,281	5.83	1,619,059	5.76	2,089,760	5.91	1,376,583	5.72
Employment	Employed	19,343,930	55.83	15,312,412	54.95	19,309,900	55.2	13,291,520	55.63
	Unemployed	1,522,535	4.39	1,269,260	4.55	2,516,488	7.19	1,463,896	6.13
	Homemaker	1,901,781	5.49	1,521,438	5.46	1,668,754	4.77	1,165,173	4.88
	Student	1,296,619	3.74	1,046,187	3.75	1,285,713	3.68	80,8341	3.38
	Retired	8,152,453	23.53	6,802,932	24.41	8,107,913	23.8	5,770,455	24.15
	Unable to work	2,433,459	7.02	1,914,816	6.87	2,091,086	5.98	1,393,952	5.83
Income level	< USD 35,000	10,136,312	34.72	8,016,867	34.55	9,171,928	31.78	5,882,478	30.41
	Between USD 35,000–75,000	8,278,722	28.36	6,440,716	27.76	7,974,662	27.63	5,454,622	28.2
	> USD 75,000	10,775,406	36.91	8,746,480	37.69	11,715,830	40.59	8,005,611	41.39
Blindness	Yes, I am blind or have serious difficulty seeing	1,837,021	5.39	1,493,232	5.46	1,701,068	4.96	1,238,576	5.3
	No	32,222,873	94.61	25,875,330	94.54	32,564,365	95.04	22,140,320	94.7
High blood pressure/ diabetes testing	Yes, I have tested in the past 3 years	8,466,257	60.83	5,934,190	60.25	8,814,629	56.63	3,336,624	57.52
	No	5,452,393	39.17	3,914,326	39.75	6,751,186	43.37	2,464,414	42.48
Diabetes/ retinopathy	Yes, diabetes has affected my eyes	267,222	21.46	282,539	17.73	152,080	18.62	223,363	19.19
	No	978,174	78.54	1,311,371	82.27	664,664	81.38	940,416	80.81
Pre-diabetic	Yes, I have been told I am	1,942,238	13.52	1,337,154	13.06	2,367,021	14.68	848,396	14.04
	No	12,420,575	86.48	8,903,543	86.94	13,760,137	85.32	5,196,482	85.97
Visit diabetes doctor	More than one time in the past year	34,914,139	99.63	28,146,737	99.44	35,550,973	99.74	24,186,956	99.52
	None	131,151	0.37	157,861	0.558	93,067	0.261	115,459	0.475
Type of insurance coverage	Through employer	1,141,875	45.17	3,182,005	42.05	3,285,970	47.93	9,866,517	42.03
	Personal plan	266,208	10.53	716,526	9.47	641,013	9.35	2,152,566	9.17
	State programs (Medicare, Medicaid, etc.)	920,660	36.42	2,899,253	38.31	2,510,187	36.62	8,386,268	35.73
	Military (TRICARE, Veterans Affairs, etc.)	110,076	4.35	217,368	2.87	220,172	3.21	779,777	3.32
	Other sources	80,337	3.18	239,336	3.16	180,342	2.63	673,114	2.87
	None	9,070	0.36	313,127	4.14	17,663	0.26	1,615,760	6.88
Race/ ethnicity	White, non-Hispanic	23,735,633	67.65	19,072,793	67.26	23,519,706	65.95	15,670,625	64.38
	Black, non-Hispanic	3,363,827	9.59	2,582,056	9.12	3,225,033	9.04	2,213,461	9.09
	Hispanic	5269328	15.02	4613885	16.27	5819462	16.32	4301606	17.67
	Other	2717543	7.75	2088476	7.36	3100211	8.69	2155222	8.85
Provider of the majority of healthcare	Family/General practitioner	189065	70.94	4577	60.67	602278	72.26	164480	67.07
	Other doctors	77450	29.06	2967	39.33	231167	27.74	80766	32.93
Age	40–44 years old	2424126	7.04	1910183	6.85	2574128	7.37	1860148	7.8
	45–49 years old	2627926	7.63	2001118	7.17	2596337	7.3	1730210	7.26
	50–54 years old	3007716	8.73	2353621	8.44	2986363	8.55	2057093	8.63
	55–59 years old	3328569	9.66	2671280	9.57	3199981	9.16	2151936	9.03
	60–64 years old	3464441	10.06	2812436	10.08	3391643	9.71	2312949	9.7
	65–69 years old	3231215	9.38	2657507	9.52	3115639	8.92	2220841	9.32
	70–74 years old	2659004	7.72	2290467	8.21	2686768	7.69	1945745	8.16
	75–79 years old	1715634	4.98	1497458	5.37	1783988	5.12	1255081	5.27
	80+ years old	1796204	5.21	1542881	5.53	1797977	5.15	1293010	5.42
	Less than 40 years old	10198099	29.6	8164636	29.26	10795602	30.91	7010107	29.41
BMI	Underweight	516140	1.6	441618	1.71	543820	1.71	357223	1.64
	Normal weight	10074172	31.32	7919495	30.68	9996356	31.36	6572615	30.2
	Overweight/ obese	21575912	67.08	17453601	67.61	21339730	66.94	14830673	68.15
Language	English	32562297	92.82	25928166	91.99	33065885	92.71	22130234	90.92
	Spanish	2517165	7.18	2256398	8.01	2598510	7.29	2210679	9.08
Region classification	Urban	31,641,154	92.64	25404464	92.44	32449140	93.56	21881326	93.37
	Rural	2,513,026	7.36	2,078,146	7.56	2,232,573	6.44	1,554,658	6.63

Of the 27 selected variables, they include explanatory and the response variable (blindness).

After running a univariate analysis to pick the most significant variables, we performed a bivariate analysis of the response variable (blindness) and each of the 17 explanatory variables. From this analysis, it can be seen that the major characteristics of people who are blind/have difficulty seeing are those that are from the South, their general health is fair/poor, have had checkups in the past 2 years, have never been told that they have diabetes, graduated high school, make < USD 35,000 a year, have state health insurance, are white/non-Hispanic, are overweight/obese, speak English, and live in an urban area. These results are shown in Table 3.

**Table 3:** The bivariate survey logistic regression of the response variable (blindness) and each explanatory variable by year.

**Variable**		**2018 (*****n*** = **1,837,021)**			**2019 (*****n*** = **1,493,232)**			**2020 (*****n*** **=** **1,701,068)**			**2021 (n** = **1,238,576)**		
		**Total people from the survey**	**# of those who answered yes to blind variable**	**Proportion of blind people (%)**	**Total people from the survey**	**# of those who answered yes to blind variable**	**Proportion of blind people (%)**	**Total people from the survey**	**# of those who answered yes to blind variable**	**Proportion of blind people (%)**	**Total people from the survey**	**# of those who answered yes to blind variable**	**Proportion of blind people (%)**
Region	Northeast	5,020,880	205,247	4.09	3,66,2703	151,542	4.14	5,390,689	200,357	3.72	3,628,617	162,550	4.48
	Midwest	8,180,076	358,940	4.39	6,54,8514	260,361	3.98	7,160,230	283,926	3.97	5,133,029	192,884	3.76
	South	12,681,643	736,326	5.81	10,204,953	612,774	6.01	11,643,231	642,359	5.52	7,327,319	415,130	5.67
	West	8,271,581	361,638	4.37	7,066,440	312,940	4.43	10,487,563	383,641	3.66	7,347,019	319,179	4.34
	Guam, Puerto Rico	932,151	174,871	18.76	874,602	155,815	17.82	982,698	190,784	19.41	904,930	148,833	16.45
Sex	Male	16,688,903	774,348	4.64	13,485,224	617,137	4.58	17,043,091	725,643	4.26	11,707,118	525,104	4.49
	Female	18,322,938	1,056,216	5.76	14,871,987	876,095	5.89	18,621,321	975,424	5.24	12,633,795	713,471	5.65
General Health	Excellent, very good	17,440,893	370,813	2.13	13,887,471	318,017	2.29	20,115,955	444,342	2.21	12,684,965	299201	2.36
	Good	11,057,040	519,708	4.7	9,000,998	408,404	4.54	10,329,572	530,075	5.13	7,614,492	370,348	4.86
	Fair, poor	6,505,269	936,912	14.4	5,403,937	759,253	14.05	5,146,077	7,18,830	13.97	3,985,216	563,261	14.13
Personal Doctor	Yes, I have 1+doctors providing health care	27,846,983	1,489,260	5.35	22,525,029	1,203,107	5.34	28,192,581	1,386,580	4.92	20,605,467	1,064,774	5.17
	No	7,024,489	335,665	4.78	5,697,952	2,79,015	4.9	7,285,537	3,04,073	4.17	3,526,172	161,823	4.59
Medical cost	Yes, I have not seen a doctor due to cost	4,183,786	456,735	10.92	3,494,096	3,84,976	11.02	3,428,567	3,25,276	9.49	2,143,989	2,35,897	11
	No	30,747,952	1,369,047	4.45	24,787,186	1,099,010	4.43	32,149,947	1,365,252	4.25	22,126,009	997,532	4.51
Checkups	I had a checkup within the past 2 years	30,784,216	1,628,862	5.29	24,963,834	1,318,747	5.28	31,410,602	1,506,219	4.8	21,293,014	1,101,697	5.17
	Has been more than 5+years or never had one	3,649,613	1,68,275	4.61	2,905,854	146,302	5.03	3,649,868	158,399	4.34	2,593,966	110,287	4.25
Hypertension Medication	Yes, I take high blood pressure medication	N/A	N/A	N/A	8,15,8078	654,071	8.02	N/A	N/A	N/A	6,978,901	5,72,720	8.21
	No	N/A	N/A	N/A	2,093,590	139,573	6.67	N/A	N/A	N/A	1,688,434	103,697	6.14
Diabetes	Yes, I have been told I have diabetes	4,705,499	5,00,496	10.64	3,783,200	4,06,507	10.75	4,478,324	459,463	10.26	3,216,587	351,384	10.92
	No, I have never been told	30,316,423	1,328,665	4.38	24,517,615	1,080,080	4.41	31,113,520	1,234,702	3.97	21,072,074	881,129	4.18
Education	Less than high school	1,119,418	157,215	14.04	1,011,138	149,352	14.77	1,053,100	147,636	14.02	844,407	123,183	14.59
	Graduated high school	10,457,595	814,219	7.79	8,276,293	621,869	7.51	9,957,358	702,851	7.06	6,628,012	486,261	7.34
	Attended college	9,455,757	495,974	5.25	7,599,214	4,08,666	5.38	9,382,514	480,327	5.12	6,213,126	348,596	5.61
	Graduated college	13,918,884	362,718	2.61	11,342,942	3,071,79	2.71	15,090,952	362,504	2.4	10,518,491	274,667	2.61
Employment status	Employed	19,343,930	526,058	2.72	15,312,412	455,058	2.97	19,309,900	518,455	2.68	13,291,520	361,075	2.72
	Unemployed	1,522,535	12,6048	8.28	1,269,260	101,758	8.02	2,516,488	152,839	6.07	1,463,896	109,467	7.48
	Homemaker	1,901,781	129,196	6.79	1,521,438	110,268	7.25	1,668,754	102,610	6.15	1,165,173	87,788	7.53
	Student	1,296,619	33,454	2.58	1,046,187	29,516	2.82	1,285,713	38,228	2.97	8,08,341	22,423	2.77
	Retired	8,152,453	509,399	6.25	6,802,932	418,411	6.15	8,107,913	516,609	6.37	5,770,455	374,880	6.5
	Unable to work	2,433,459	499,197	20.51	1,914,816	363,053	18.96	2,091,086	357,561	17.1	1,393,952	270,484	19.4
Income status	Less than USD 35,000	10,136,312	1,020,618	10.07	8,016,867	812,126	10.13	9,171,928	865,259	9.43	5,882,478	620,092	10.54
	Between USD 35,000 and 74,999	8,278,722	2,93,927	3.55	6,440,716	239,456	3.72	7,974,662	278,323	3.49	5,454,622	222,072	4.07
	Greater than USD 75,000	10,775,406	1,79,954	1.67	8,746,480	153,433	1.75	11,715,830	190,220	1.62	8,005,611	139,523	1.74
Primary source of health insurance	Through employer	1,141,875	27,203	2.38	3,182,005	60,162	1.89	3,285,970	62,764	1.91	9,866,517	205,076	2.08
	Personal plan	266,208	13,087	4.92	7,16,526	28,398	3.96	641,013	16,484	2.57	2152566	84212	3.91
	State programs (Medicare and Medicaid.)	9,20,660	93,326	10.14	2,899,253	230,353	7.95	2,510,187	185,905	7.41	8,386,268	710713	8.47
	Military (TRICARE, Veterans Affairs, etc.)	110,076	5,488	4.99	217,368	10,591	4.87	220,172	13,498	6.13	779,777	45,358	5.82
	Other sources	80,337	8,205	10.21	239,336	14,074	5.88	180,342	11,229	6.23	673,114	42,174	6.27
	None	9,070	1,308	14.42	313,127	18,971	6.06	17,663	402.05	2.28	1,615,760	104,683	6.48
Race/ Ethnicity	White, non-Hispanic	23,735,633	9,79,986	4.13	19,072,793	7,75,201	4.06	23,519,706	863,478	3.67	15,670,625	620,051	3.96
	Black, non-Hispanic	3,363,827	2,33,987	6.96	2,582,056	1,91,609	7.42	3,225,033	205,162	6.36	2,213,461	144,538	6.53
	Hispanic	5,269,328	4,67,032	8.86	4,613,885	4,16,080	9.02	5,819,462	497,451	8.55	4,301,606	375738	8.73
	Other	2,717,543	156,017	5.74	2,088,476	110,343	5.28	3,100,211	134,977	4.35	2,155,222	98248	4.56
Age	40–44 years old	2,424,126	86,463	3.57	1,910,183	65,394	3.42	2,574,128	83,031	3.23	1,860,148	68308	3.67
	45–49 years old	2,627,926	134,505	5.12	2,001,118	109,376	5.47	2,596,337	123,268	4.75	1,730,210	8,6292	4.99
	50–54 years old	3,007,716	2,01,948	6.71	2,353,621	144,881	6.16	2,986,363	174,494	5.84	2,057,093	119,646	5.82
	55–59 years old	3,328,569	2,28,505	6.86	2,671,280	1,85,775	6.95	3,199,981	181,124	5.66	2,151,936	135,743	6.31
	60–64 years old	3,464,441	223,269	6.44	2,812,436	174,490	6.2	3,391,643	196,528	5.79	2,312,949	148,958	6.44
	65–69 years old	3,231,215	196,726	6.09	2,657,507	154,210	5.8	3,115,639	163,771	5.26	2,220,841	139,522	6.28
	70–74 years old	2,659,004	1,64,890	6.2	2,290,467	133,231	5.82	2,686,768	162,475	6.05	1,945,745	113,403	5.83
	75–79 years old	1,715,634	121,241	7.07	1,497,458	106,571	7.12	1,783,988	113,081	6.34	1,255,081	84,878	6.76
	80+ years old	1,796,204	165,189	9.2	1,542,881	145,716	9.44	1,797,977	182,235	10.14	1,293,010	138,708	10.73
	18–39 years old	10,198,099	2,92,749	2.87	8,164,636	259,544	3.18	1,079,5602	300,381	2.78	7,010,107	189,487	2.7
BMI	Underweight	516,140	41,081	7.96	441,618	34,674	7.85	543,820	38,119	7.01	357,223	23,986	6.71
	Normal weight	10,074,172	4,78,934	4.75	7,919,495	375,465	4.74	9,996,356	419,133	4.19	6,572,615	296,284	4.51
	Overweight/obese	21,575,912	1,198,227	5.55	17,453,601	989,140	5.67	21,339,730	1,122,217	5.26	14,830,673	825,193	5.56
Language	English	32,562,297	1,520,078	4.67	25,928,166	1,198,654	4.62	33,065,885	1,367,991	4.14	22,130,234	967,473	4.37
	Spanish	2,517,165	316,701	12.58	2,256,398	283,668	12.57	2,598,510	333,077	12.82	2,210,679	271,103	12.26
Urban/ Rural Class	Urban	31,641,154	1,502,207	4.75	25,404,464	1,212,152	4.77	32,449,140	1,378,723	4.25	21,881,326	995,761	4.55
	Rural	2,513,026	159,943	6.36	2,078,146	125,465	6.04	2,232,573	131,561	5.89	1,554,658	93,982	6.05

This includes the total number of people in the survey who answered the survey question, followed by the number of people who answered “yes” to the blind variable (“are you blind or do you have serious difficulty seeing, even when wearing glasses?”). The final column is the proportion, in percentage, of those who responded yes from the total number of people who answered that survey question.

**Figure 2:**
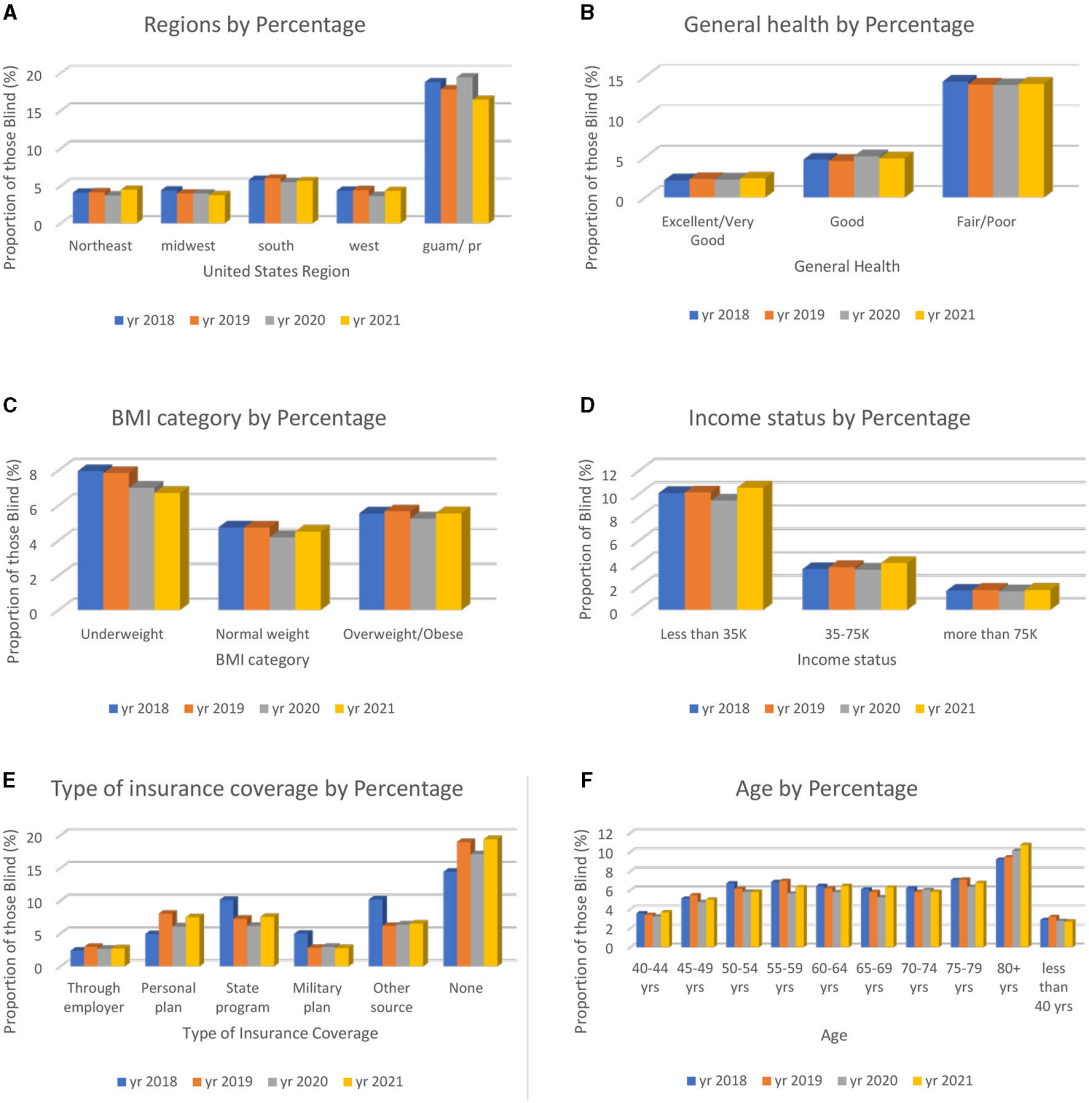
**Six** explanatory variables chosen from the bivariate analysis to show the characteristics of those who answered “yes” to blindness or having serious difficulty seeing, even with glasses. The total percentages of the combined data (2018–2021) are presented. **(A)** Total percentage of those who are VI based on geographic location state. **(B)** Total percentage of those who are VI based on personal health rating. **(C)** Total percentage of those who are VI based on BMI categories. **(D)** Total percentage of those who are VI based on income status. **(E)** Total percentage of those who are VI based on insurance source type. **(F)** Total percentage of those who are VI based on age.

In Figure 2, six explanatory variables were chosen to illustrate the importance of looking at the characteristics of those who are VI. From this analysis, we can infer which groups are more likely to have VI based on those who answered the Centers for Disease Control and Prevention BRFSS. The variables selected were “state,” “general health,” “BMI,” “income status,” “type of insurance,” and “age”.

For the multiple logistic regression analysis (Table 4), the reference group for each explanatory variable was as follows: “state”: Northeast, “sex”: male, “general health”: excellent/very good, “personal doctor”: no, “medical cost”: yes, “checkups”: never/ within past 5+ years, “diabetes”: yes, “education level”: less than high school, “employment”: student, “income level”: >75,000 a year, “type of insurance coverage”: military, “race/ethnicity”: Black, “age”: < 40 years old, “BMI”: underweight, “language”: Spanish, and “region classification”: rural. This analysis included point estimates, 95% confidence intervals, and *p*-values to show the association between the explanatory variables and the response variables based on the various levels in the explanatory variables. The significance level of *p* < 0.05 was used to determine significance in this study.

**Table 4:** Multiple logistic regression analysis of explanatory variables in association with the response variable (blindness).

**Comparison**	**Point estimate**	**95% Confidence interval**	***P-*Value**
State: Midwest vs. Northeast	1.03	0.826–1.284	0.793
State: South vs. Northeast	1.611	1.324–1.960	< 0.0001^*^
State: West vs. Northeast	1.155	0.932–1.432	0.189
Sex: female vs. male	1.099	0.981–1.232	0.105
General Health: fair/ poor vs. excellent/ very good	2.923	2.475–3.452	< 0.0001
General Health: good vs. excellent/very good	1.521	1.297–1.784	< 0.0001
Personal doctor: yes, one or more vs. no provider	0.791	0.664–0.941	0.008
Medical cost: no vs. yes	0.592	0.512–0.685	< 0.0001
Checkups: within past 2 years vs. never/5+ years	0.936	0.730–1.201	0.604
Diabetes: no vs. yes	0.842	0.739–0.959	0.010
Education level: graduated high school vs. less than high school	0.928	0.724–1.189	0.555
Education level: attended college vs. less than high school	0.833	0.642–1.081	0.170
Education level: graduated college vs. less than high school	0.645	0.489–0.850	0.002
Employment status: employed vs. student	1.507	0.916–2.480	0.107
Employment status: unemployed vs. student	2.86	1.662–4.919	0.0001
Employment status: homemaker vs. student	1.854	1.076–3.197	0.026
Employment status: retired vs. student	1.774	1.048–3.003	0.033
Employment status: unable to work vs. student	3.641	2.156–6.148	< 0.0001
Income status: < USD 35,000 vs. > USD 75,000	1.683	1.347–2.102	< 0.0001
Income status: Between USD 35,000–75,000 vs. > USD 75,000	1.157	0.930–1.439	0.190
Type of health insurance: no plan vs. military plan	2.517	1.288–4.920	0.007
Type of health insurance: through employer vs. military plan	0.86	0.648–1.142	0.299
Type of health insurance: personal plan vs. military plan	1.092	0.800–1.492	0.579
Type of health insurance: state program vs. military plan	1.409	1.089–1.822	0.009
Type of health insurance: another source vs. military plan	1.638	1.160–2.313	0.005
Race/ethnicity: Other vs. Black	1.154	0.900–1.481	0.259
Race/ethnicity: White vs. Black	0.833	0.714–0.971	0.020
Race/ethnicity: Hispanic vs. Black	1.167	0.893–1.526	0.258
Age: less than 40 years vs. 40–44 years	1.126	0.801–1.583	0.495
Age: 45–49 years vs. 40–44 years	2.069	1.460–2.934	< 0.0001
Age: 50–54 years vs. 40–44 years	2.318	1.644–3.269	< 0.0001
Age: 55–59 years vs. 40–44 years	1.971	1.414–2.747	< 0.0001
Age: 60–64 years vs. 40–44 years	1.701	1.214–2.383	0.002
Age: 65–69 years vs. 40–44 years	1.833	1.290–2.604	0.001
Age: 70–74 years vs. 40–44 years	1.752	1.224–2.507	0.002
Age: 75–79 years vs. 40–44 years	2.186	1.501–3.183	< 0.0001
Age: 80+ years vs. 40–44 years	3.604	2.514–5.167	< 0.0001
BMI: normal weight vs. underweight	1	0.678–1.474	0.999
BMI: overweight/obese vs. underweight	0.912	0.623–1.334	0.634
Language: English vs. Spanish	0.7	0.465–1.055	0.088
Region classification: urban vs. rural	0.87	0.756–1.002	0.053

This includes point estimates (odds ratios), 95% confidence intervals, and p-values.

The factors listed above have an association with blindness or having serious difficulty seeing based on their odds ratio. When the odds ratio is higher than 1, that factor is considered a risk factor. The descriptive and bivariate and multivariate regression analyses provide a key understanding of the characteristics of someone who is blind or has serious difficulty seeing, even when wearing glasses. We gathered the frequencies, percentages, confidence intervals, point estimates, and *p*-values to gain a full understanding of the socioeconomic status, demographics, and health status of individuals who answered “yes” to the question “are you blind or do you have serious difficulties seeing, even when wearing glasses?”

## Discussion

There have been studies on eye health and the factors affecting the utilization of eye care services. The evidence from previous studies suggests that VI is associated with a low quality of life and compromised physical, emotional, and social wellbeing (1, 2, 11). Another study found that ophthalmic health is associated with SDOHs and that VI is associated with many disparities in eye care (education, genetics, etc.) and barriers to health care access (transportation, reliable doctors, and income) (5). These studies show that social determinants (race, income, geographic location, sex, employment, and healthcare coverage source) have a large impact on the quality of eye care services and the access to eye care services and health as they all have found a significant relationship to VI. Groups including racial and ethnic minorities, low SES individuals, and uninsured individuals receive ophthalmic screening, preventative care, and treatments at lower rates and lower standards (5). Race and ethnicity play a huge role in the type of health care one may receive, yet minorities and marginalized individuals are more susceptible to diseases that can, in turn, impact their eye health if left untreated. Halawa et al. (6) discussed the barriers to access to health care for racial minorities which are low levels of health insurance coverage, geographic locations, access to high quality care, and systemic racial differences in care. People who access proper care encounter other barriers such as high treatment costs, the need for regular follow-ups, and interactions with other health sectors (financial, pharmaceutical, providers, and counseling), all of which reduce overall patient cooperation and compliance (attending follow-ups and taking medication properly) (1).

We must find a way to create better treatment options and make follow-up care accessible. To treat those with VI, it is important to do so at the early stages to reduce the severity of vision loss. Comprehensive eye care (CEC) is a holistic new approach that encompasses treatment, prevention, promotion, and rehabilitation for blindness (1). CEC is a way to decrease the number of people who are diagnosed with an eye disease causing blindness or progressive vision loss. To this end, we need to educate patients and physicians on the comorbidities, groups who have higher chances of developing eye diseases, and available interventions depending on the disease such as cataract surgery, corrective lenses, and anti-VEGF therapy, which have been shown to improve the quality of life for 150 million people worldwide (2). To implement these interventions, we must encourage people to undergo annual screenings regardless of whether or not experiencing visual changes. More financial support should be spent on education for the public. The U.S. spends most of its funds on pharmaceuticals, high-volume/high-margin procedures, CT and MRI imaging, and administration costs that makeup two-thirds of the difference in healthcare costs between the U.S. and other developed countries (18). If the spending was minimized in these four areas, we could then create a more preventative initiative, such as pop-up clinics, to allow minorities and those in the low SES to obtain free and easily accessible eye screenings, which could decrease the rate of vision loss in the U.S. and make us a healthier nation.

It is critical to prevent the occurrence of diseases that can impact eye health by ensuring that all social groups are equally educated about them. This can be one of the only ways to truly decrease the number of people who lose vision due to health issues that could have been prevented by increasing the level of health literacy. All social groups should be equally educated about diseases they have that can affect the health of the eyes, as well as the characteristics/factors that can increase the risk of developing eye diseases. Health literacy is delivered in many forms including print literacy (written information), oral literacy (verbally informed), and numeracy (statistical data) (3). Multiple forms need to be available to reach every person in a way that can be informative. For example, a blind individual cannot read information unless it is braille so they must be able to listen to it, or someone who is deaf needs to be able to read the information. In the U.S. 76% of adults have an education level less than a high school degree and 59% of older adult (65+ years old) are below or at the base level (3). We must make efforts to reach all individuals so there are no disparities in health education based on race, education level, age, and geographic location. It has been found that “low health literacy is associated with increased hospitalizations, increased emergency care use, lower rates of mammography and influenza vaccination, decreased ability to take medications appropriately, decreased ability to understand labels, and higher mortality among older adult” (3) patients and physicians. We can address these issues and foster a healthier and more knowledgeable society.

The multiple logistic regression analysis identified some risk factors that have a significant effect on the occurrence of the disease: coming from a Southern state (OR 1.611 *p* < 0.0001 CI 1.324–1.960), general health classified as fair/poor (OR 2.923 *p* < 0.0001 CI 2.475–3.452), employment status of unable to work (OR 1.683 *p* < 0.0001 CI 1.347–2.102), income status of less than USD 35,000 a year (OR 1.683 *p* < 0.0001 CI 1.347–2.102), no insurance coverage (OR 2.517 p 0.0069 CI 1.288–4.920), and between the ages of 50–54 years old (OR 2.318 *p* < 0.0001 CI 1.644–3.269), the data are shown in Table 5. From this analysis, we identified six variables that are known to be relevant in those who are blind/have difficulty seeing. The odds ratios were not only < 1 but the *p*-values were significant as α was >0.05 and the confidence intervals did not include 1. We found significant variables that define the high-risk population and indicate the characteristic traits of those who are more vulnerable to suffering from vision loss.

**Table 5:** The explanatory variables are significantly associated with VI.

**Variables that were found to be associated with VI**	
**Variable**	**The odds ratios of VI**
**U.S. Region**	
Northeast (ref)	1.00
Midwest	1.03
South	1.61
West	1.1
**General health perception**	
Excellent/very good (ref)	1.00
Good	1.52
Fair/poor	2.92
**Employment status**	
Student (ref)	1.00
Employed	1.51
Unemployed	2.86
Homemaker	1.85
Retired	1.77
Unable to work	3.64
**Income status**	
< 35,000 USD	1.68
35–75,000 USD	1.16
>75,000 USD (ref)	1.00
**Age (years)**	
40–44 (ref)	1.00
45–49	2.07
50–54	2.31
55–59	1.97
60–64	1.70
65–69	1.83
70–74	1.75
75–79	2.19
80+	3.60
< 40	1.13
**Health insurance source**	
Military (ref)	1.00
No plan	2.52
Through employer	0.86
Personal plan	1.09
State program	1.41
Other sources	1.64

The reference groups are marked and have an odds ratio of 1.

BRFSS is not without limitations. Sampling bias, stemming from non-response and under coverage of certain population groups, alongside reliance on self-reported data, introduces potential sources of error. Furthermore, the cross-sectional nature of the survey restricts its ability to elucidate temporal trends or establish causal relationships. Additionally, the survey's scope may not fully capture all determinants of health outcomes, necessitating caution in interpretation. Acknowledging these limitations highlights the importance of complementing BRFSS data with other research methodologies and data sources to obtain a more nuanced understanding of public health dynamics.

## Conclusion

**Figure 3:**
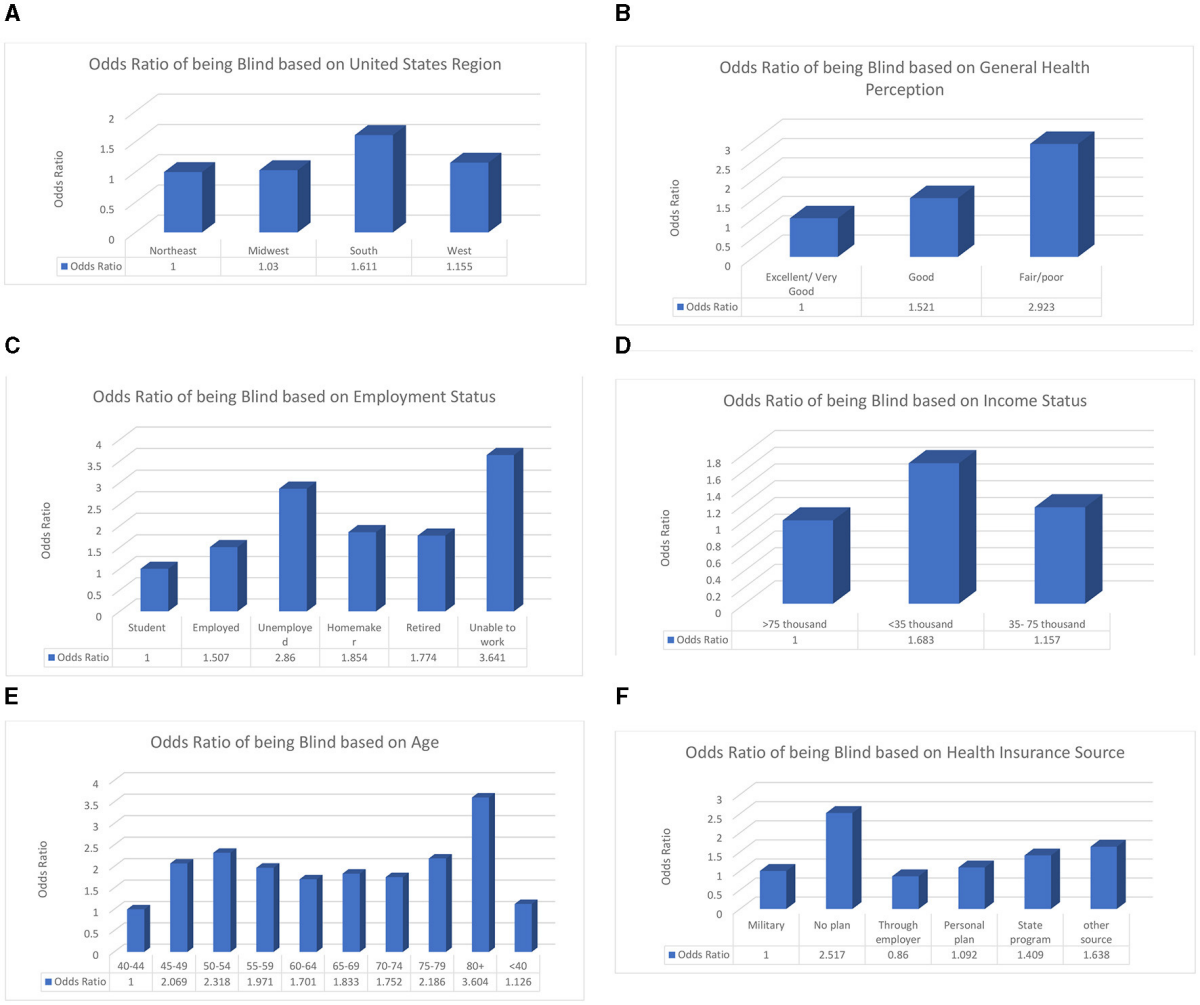
The explanatory variables are significantly associated with having difficulty seeing or being blind. The reference groups are shown as the first bar in each graph and have an odds ratio of 1. **(A)** Odds ratio of having visual impairments based on geographic location. **(B)** Odds ratio of having visual impairments based on general health perception. **(C)** Odds ratio of having visual impairments based on employment status. **(D)** Odds ratio of having visual impairments based on income status. **(E)** Odds ratio of having visual impairments based on age. **(F)** Odds ratio of having visual impairments based on health insurance source.

In this study, geographical location, perceived health status, employment status, income status, source of health insurance, and age of the individual were associated with blindness or having serious difficulties seeing. Those who live in Southern states, with no insurance, have an income level below poverty, and are of retirement age have the highest odds of suffering from VI. The factors listed above have an association with blindness or having serious difficulty seeing based on their odds ratio. When the odds ratio is higher than 1, that factor is considered a risk factor. The odds ratios are shown in Figure 3. We must study these factors further to learn the exact causal pathways that can influence whether a person becomes blind or has serious difficulty seeing so that we can reduce the number of people who suffer from visual impairment. These risk factors can guide the future development of interventions by ensuring that the individuals in risk groups are targeted along with the rest of the population.

These odds ratios show that certain groups are at a higher risk of visual impairment due to socioeconomic, biological, or demographic factors, as well as other factors that need to be examined. Future studies should delve more in depth into how medical costs, education status, and body mass index vary among at-risk populations based on different levels. Another important direction is investigating the coverage provided by the main insurance companies regarding eye care in terms of examinations, procedures, medications, regulations, and deductibles. This study showed that patients using government insurance have a greater chance of blindness than those who have private insurance.

## Data availability statement

The original contributions presented in the study are included in the article/supplementary material, further inquiries can be directed to the corresponding author.

## Author contributions

AP: Writing—review & editing, Investigation, Software. KA: Writing—review & editing, Conceptualization, Data curation, Formal analysis, Funding acquisition, Methodology. DP: Formal analysis, Investigation, Methodology, Project administration, Software, Supervision, Writing—review & editing. MD: Formal analysis, Software, Validation, Writing—review & editing. CF: Data curation, Formal analysis, Investigation, Software, Supervision, Writing—review & editing.
